# A Systematic Review on Molecular Toxicology and Omics-Based Risk Assessment of Pigments Used in Dermal Implantation Procedures: Implications for Somatology and Somatic Therapy Practice

**DOI:** 10.3390/ijms27125422

**Published:** 2026-06-16

**Authors:** Baatile Komane, Thobile Kaye, Betty Chauke, Rueben Mahlakwana

**Affiliations:** Department of Human Movement and Therapeutic Sciences, Somatology, Arcadia Campus, Tshwane University of Technology, Pretoria 0001, South Africa; kayetan@tut.ac.za (T.K.); chaukebetty@yahoo.com (B.C.); noureuben@gmail.com (R.M.)

**Keywords:** pigment implantation, molecular toxicology, omics-based risk assessment, oxidative stress, NF-κB/MAPK signalling, type IV hypersensitivity, dermal-to-lymphatic transport, nanoparticle toxicity, somatology, somatic therapy

## Abstract

Pigment implantation (semi-permanent make-up, microblading and cosmetic tattooing) introduces complex pigment mixtures into the dermis, resulting in direct exposure of keratinocytes, fibroblasts and resident immune cells to metals, organic dyes and nanoparticles. Within Somatology and Somatic therapy practice, an allied health discipline concerned with evidence-based care of the skin and body, Somatic Therapists operate at the interface of dermal intervention, molecular exposure and occupational health, underscoring the relevance of mechanistic toxicology for risk-informed professional practice. This PRISMA-guided systematic review synthesises molecular toxicology and omics-based evidence, emphasising oxidative stress generation, inflammatory signalling via NF-κB/MAPK pathways, apoptosis and genotoxicity, T-cell-mediated type IV hypersensitivity reactions associated with modern red azo pigments, and dermal-to-lymphatic transport of particulate matter. Transcriptomic and metabolomic studies consistently demonstrate pigment-specific inflammatory responses and wound-healing gene signatures, supporting mechanism-driven biocompatibility profiling. Regulatory frameworks, including EU REACH Annex XVII Entry 75 and recent FDA guidance on microbial contamination, have strengthened compliance requirements; however, surveillance continues to identify mislabelling, restricted pigments and microbial contamination in some inks. For Somatology and Somatic therapy practice, these findings highlight the importance of evidence-based pigment selection, traceable sourcing, aseptic technique, ventilation, personal protective equipment and informed consent addressing pigment migration and delayed adverse reactions. The integration of molecular outcomes with omics technologies and regulatory oversight provides a next-generation risk assessment framework to advance safe cosmetic practice and public health.

## 1. Introduction

For consistency, the term ‘pigment implantation (PI)’ is used throughout this review to collectively refer to micropigmentation, semi-permanent make-up and permanent make-up. The terms ‘Somatology’ and ‘Somatic therapy’ are used in alignment with South African professional frameworks, with Somatic therapy referring to clinical practice within the broader discipline of Somatology.

Pigment implantation (PI), also referred to as micropigmentation or permanent make-up, is a cosmetic procedure that involves the deposition of pigments into the dermal layer of the skin to create durable aesthetic enhancements such as eyeliner, lip colour and eyebrow definition [1]. The procedure employs fine needles to introduce pigment below the epidermis, thereby bypassing the skin’s primary barrier and placing complex foreign substances directly into living tissue [2]. While this implantation confers durability, it simultaneously raises safety concerns owing to the chemical complexity of pigments, which may comprise heavy metals, metal oxide nanoparticles, azo dyes and other synthetic colourants capable of eliciting toxicological responses [3,4,5].

Within this context, Somatology and Somatic therapy represent allied health disciplines concerned with evidence-based care of the skin and body, delivered through aesthetic, therapeutic and preventative interventions that support tissue integrity, physiological function and overall wellbeing. Somatic Therapists are trained practitioners who apply scientifically informed techniques, including dermal therapies and body treatments and therefore operate at the interface of dermal intervention, molecular exposure and occupational health. As such, Somatology and Somatic therapy practice necessitate adherence to ethical standards, safety protocols and evolving regulatory frameworks to mitigate both client and practitioner risk associated with pigment implantation procedures [6].

The skin is a complex, multi-layered organ comprising the epidermis, dermis and hypodermis. The epidermis functions as a protective barrier, whereas the dermis contains blood vessels, lymphatic channels and connective tissue and constitutes the primary site of pigment deposition during implantation [7,8]. Following dermal administration, implanted pigments interact with resident immune cells, including macrophages and dendritic cells, which can internalise pigment particles and facilitate their transport along lymphatic pathways to regional lymph nodes and, in some cases, to secondary organs [2,9,10,11,12]. These cellular and biokinetic processes underpin growing concerns regarding pigment persistence, lymphatic redistribution and sustained immunological engagement following pigment implantation.

The integration of toxicology with Somatology/Somatic therapy practice is essential to advance pigment safety and align with Sustainable Development Goal 3 on health and wellbeing. In South Africa, the Allied Health Professions Council of South Africa (AHPCSA) provides statutory oversight for allied health professions and has initiated processes concerning Somatology’s inclusion on the register, emphasising public protection, ethical practice and safety standards [12,13,14]. Incorporating AHPCSA guidance into pigment safety protocols supports evidence-based approaches, minimises toxicological risks and promotes occupational health measures including personal protective equipment (PPE), ventilation and sterilisation protocols [15]. This alignment improves consumer confidence and positions Somatology/Somatic therapy within a global framework for ethical, safe and sustainable cosmetic practice.

This systematic review, conducted in accordance with PRISMA 2020 guidance, synthesises evidence on pigment composition, toxicological mechanisms, omics technologies and regulatory frameworks, while exploring implications for Somatology, Somatic therapy and occupational health standards [16,17]. Key findings across the literature include frequent non-compliance with compositional limits (notably nickel and chromium (VI)), the mislabelling of ingredients, occasional microbial contamination of commercial inks, dermal-to-lymphatic migration of nanoparticles such as titanium dioxide and carbon-based particles and diverse hypersensitivity reactions, particularly with red azo pigments [3,4,5,10,11,18,19,20,21,22]. EU REACH Annex XVII Entry 75 now restricts hazardous substances in tattoo and permanent make-up inks and introduced phased timelines for specific pigments including Pigment Blue 15:3 and Pigment Green 7; parallel proposals are progressing under UK REACH [23,24,25,26].

**Figure 1:**
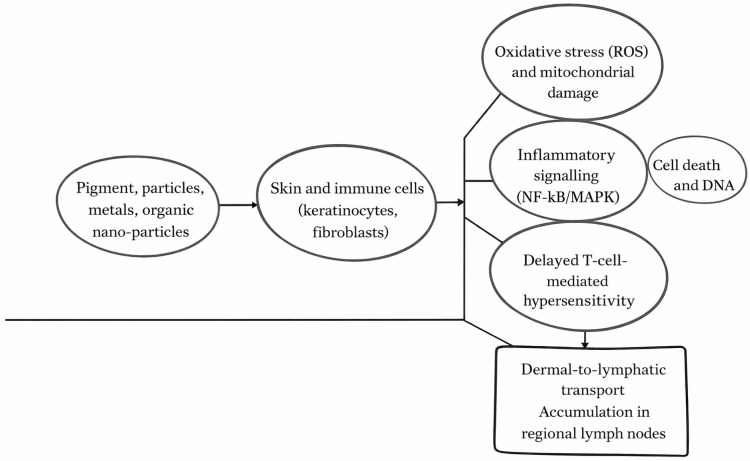
Mechanistic toxicology pathway for dermal pigment implantation.

The molecular framing of dermal pigment exposure depicted in Figure 1 provides a guideline on how pigment implantation bypasses the stratum corneum, placing particles directly into the dermis where keratinocytes, fibroblasts, macrophages and dendritic cells engage foreign materials. Nanoparticle size, surface chemistry and pigment composition govern cellular uptake, reactive oxygen species generation and the activation of NF-κB/MAPK signalling, resulting in IL-8 and other cytokine release, apoptosis and genotoxic stress [4,5,27,28,29,30,31,32]. Dermal macrophage-engulfed and dendritic cell migration underpin rapid lymphatic transport to regional nodes [10,11]. In addition, oxidative stress with ROS and mitochondrial dysfunction; NF-κB/MAPK inflammatory signalling; apoptotic and genotoxic responses; type IV T-cell hypersensitivity; and xenobiotic metabolism dysregulation for PAHs and azo dyes couple to dermal-to-lymphatic transport via macrophage/dendritic cell migration [3,4,5,10,27,28,29,30,31,32].

Standardised transcriptomic and metabolomic pathway-specific responses can reveal ink-specific inflammatory trajectories (such as differences between black and red exposures) and wound-healing gene programmes, offering reproducible molecular markers for biocompatibility profiling and safer-by-design pigment selection. Integration with xenobiotic metabolism pathways (e.g., PAH and azo dye breakdown to aromatic amines) strengthens causal inference in risk assessment [3,4,20,30].

Rationale:

The increasing popularity of PI has amplified concerns regarding pigment safety, particularly the potential for local and systemic toxicity, allergic reactions, and long-term health effects. The existing literature highlights gaps in regulatory frameworks and the need for advanced toxicological assessments that incorporate omics technologies for mechanistic insights [6].

Research question:

What are the toxicological risks associated with semi-permanent make-up pigments and how can omics technologies and Somatology/Somatic therapy principles be integrated to improve safety standards?

Objectives:

1. To review the composition and toxicological mechanisms of pigments used in PI.
2. To evaluate the role of omics technologies in advancing pigment safety assessment.
3. To examine regulatory frameworks and identify gaps.
4. To explore the integration of Somatology/Somatic therapy principles in promoting safe cosmetic practices.

## 2. Methodology

**Figure 2:**
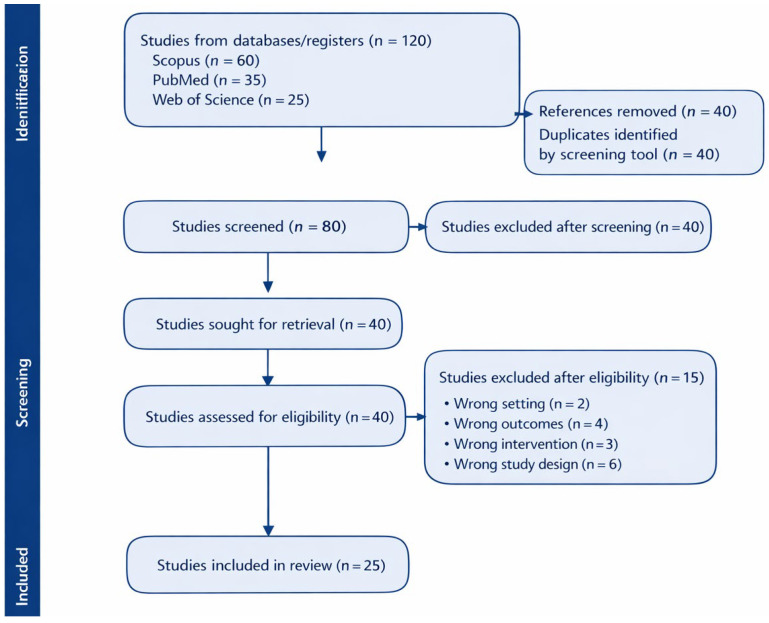
PRISMA flow diagram of study selection. Link https://www.crd.york.ac.uk/PROSPERO/view/CRD420261283033 (accessed on 11 January 2026).

This systematic review was conducted in accordance with the PRISMA 2020 guidelines to ensure transparency, reproducibility and methodological rigour (Figure 2) [16,17]. The review protocol was prospectively registered with PROSPERO (CRD42025128303).

### 2.1. Literature Search Strategy

A comprehensive literature search was performed in PubMed, Scopus and Web of Science to identify relevant studies addressing pigment implantation, toxicological mechanisms, molecular and omics-based evidence, Somatology practice, occupational health and safety (OHS) and regulatory compliance [16,17]. Searches were supplemented with regulatory and grey-literature sources including the European Chemicals Agency (ECHA) and the United States Food and Drug Administration (FDA). The final search was completed in November 2025.

Table S1 depicts the search strategy’s combined controlled vocabulary and free-text terms as follows:

(“pigment implantation” OR “semi-permanent makeup” OR “micropigmentation”) AND

(“pigment toxicity” OR “heavy metals” OR “nanoparticles”) AND

(“omics technologies” OR “toxicogenomics”).

Search limits were restricted to English-language publications published between 2000 and 2025.

### 2.2. Study Selection

After duplicate removal, 120 records were identified. Title and abstract screening resulted in 80 records for further evaluation. Full-text assessment was conducted for 40 studies against predefined eligibility criteria. A total of 25 studies met the inclusion criteria and were included in the qualitative synthesis [16,17]. The study selection process is summarised in Figure 2.

The selection process included two reviewers who independently screened titles and abstracts; discrepancies were resolved by consensus [16,17]. Table 1 provides the study characteristics including year, design, pigment type used and the findings.

### 2.3. Eligibility Criteria

Eligible studies investigated pigment implantation safety, toxicological or molecular mechanisms, omics-based outcomes, regulatory frameworks or occupational health considerations. Experimental designs included in vitro, in vivo, mechanistic and omics-based studies, together with reviews, case reports and regulatory documents. Studies unrelated to pigment toxicity, lacking toxicological outcomes or not published in English were excluded.

### 2.4. Occupational Health and Safety Considerations

Although OHS was not a primary outcome, it was incorporated into the eligibility criteria and interpretive synthesis to address practitioner safety in pigment implantation procedures. Included studies were examined for reporting on PPE use, ventilation systems, sterilisation protocols and measures to mitigate occupational exposure to hazardous substances such as heavy metals and nanoparticles [6,7].

### 2.5. Data Extraction and Risk of Bias Assessment

Data were extracted using a predefined template capturing study design, pigment composition, toxicological mechanisms, molecular or omics findings, regulatory compliance and OHS measures; the template is depicted in Table S2 [8]. Risk of bias was assessed using tools adapted for in vitro, in vivo and mechanistic studies, evaluating selection, performance and detection bias [1,2,3,4,5]. Overall risk of bias was low to moderate across studies, with low risk due to missing results. Certainty of evidence was rated as moderate using GRADE-informed principles (Table 2).

This methodological framework was chosen to systematically evaluate molecular toxicological evidence alongside regulatory and occupational health data, supporting evidence-based translation to Somatology and Somatic therapy practice.

## 3. Results

The PRISMA flow diagram (Figure 2) summarises study selection. After duplicate removal, 120 records were identified, 80 were screened by title and abstract, 40 underwent full-text assessment and 25 met the eligibility criteria for qualitative synthesis [16,17].

### 3.1. Pigment Composition and Contamination

Across included studies, pigments typically contained metal oxides and nanoparticles together with organic colourants [2]. CuO and high-dose ZnO showed dose-dependent cytotoxicity in keratinocytes and fibroblasts, whereas TiO_2_ and Fe_2_O_3_ showed lower cytotoxicity under the reported conditions [4]. Ex vivo human skin and 3D tattooed skin models demonstrated dermal deposition, proximity to dermal vessels, apoptosis and IL-8 upregulation, with red formulations most frequently implicated [4,8]. Market and analytical surveys reported undeclared additives and labelling inaccuracies (including restricted pigments Pigment Green 7 and Pigment Blue 15:3) in some products [2,3].

Microbiological surveillance by regulators identified contamination in commercial inks, including findings in sealed bottles and documented voluntary recalls over two decades [11]; targeted 2025 alerts also identified *Pseudomonas aeruginosa* contamination in specific lots [12].

### 3.2. Dermal-to-Lymphatic Migration and Biokinetics

Preclinical studies showed rapid pigment drainage to regional lymph nodes, persistence over months, macrophage death and sustained inflammation in draining nodes [5]. Human tracer work (Tat_BioV) quantified systemic exposure following tattooing, with urine and plasma detection within 24 h, and indicated skin-specific first-pass metabolism for soluble constituents; a worst-case exposure of 0.31 g ink per session (75th percentile) was estimated [6]. Epidemiological analyses reported that signals for lymphoma and skin cancer are variably significant after adjustment; causality remains unresolved and larger longitudinal cohorts are required [5].

### 3.3. Immunological and Dermatological Reactions

Clinical sources documented acute inflammation; allergic contact dermatitis; photo-aggravated reactions (notably cadmium yellow); and granulomatous, lichenoid and pseudo-lymphomatous presentations [7]. Red pigments are disproportionately implicated: historically cinnabar/mercury sulphide and more recently red azo pigments [7,9]. Omics-enabled models consistently reported oxidative stress and inflammatory pathway activation, with wound-healing gene programmes that differ between black and red inks [8]; ex vivo studies showed apoptosis and cytokine induction together with pigment localisation near vessels [4].

### 3.4. Regulatory Frameworks and Compliance Signals

EU REACH Annex XVII Entry 75 restricts hazardous substances in tattoo and permanent make-up inks and introduced phased entry for Pigment Blue 15:3 and Pigment Green 7 [9]. UK REACH (HSE) has advanced a consolidated final opinion recommending parallel restrictions and transition periods [10]. The FDA (MoCRA) issued final guidance (2024) on insanitary conditions and microbial contamination, noting multiple illness reports, contamination in sealed bottles and 18 voluntary recalls (2003–2024) [11]; 2025 safety alerts documented *Pseudomonas*-contaminated lots [12]. A summary of representative toxicological outcomes from individual studies is provided in Table 3.

### 3.5. Nanoparticle Transport, Persistence and Cellular Interactions

Early mechanistic investigations using synchrotron-based imaging and spectroscopic approaches, particularly at the European Synchrotron Radiation Facility (ESRF), demonstrated that tattoo pigments frequently contain nanoparticle-sized fractions (<100 nm) that migrate beyond the dermal injection site. Using X-ray fluorescence microscopy and X-ray absorption spectroscopy, these studies provided direct evidence of carbon black and organometallic nanoparticles localising within regional lymph nodes, with particle size influencing transport efficiency and retention [13,14].

Nanoparticles derived from black pigments were shown to persist intracellularly within macrophages, while larger pigment aggregates remained primarily extracellular in the dermis. These findings established a mechanistic basis for chronic immune activation, long-term pigment persistence, and lymph node pigmentation observed clinically [13]. Subsequent animal studies corroborated these observations, identifying macrophage-mediated shuttling as a primary route of pigment redistribution following dermal injection [14,15].

Importantly, synchrotron analyses also demonstrated that pigment nanoparticles may undergo surface modification and partial oxidation within the biological milieu, potentially altering reactivity and toxicological behaviour over time [18]. Such transformations are consistent with later observations of immune cell stress and apoptosis following prolonged pigment exposure in lymphatic tissues.

### 3.6. Polycyclic Aromatic Hydrocarbons (PAHs) and Photochemical Activation

Complementary mechanistic toxicology studies have focused on polycyclic aromatic hydrocarbons (PAHs) present in carbon-based black inks. Quantitative chemical analyses identified benzo[a]pyrene and other high-molecular-weight PAHs at concentrations sufficient to elicit genotoxic and pro-inflammatory effects in experimental systems [19,20].

In vitro studies using human keratinocytes and fibroblasts demonstrated ROS generation, DNA strand breaks and altered xenobiotic metabolism pathways following exposure to PAH-containing ink extracts [18]. These effects were significantly amplified by ultraviolet (UV) exposure, supporting a photochemical mechanism whereby PAHs act as photo-sensitisers, generating reactive intermediates upon light absorption [19,21].

Laser irradiation, commonly used in tattoo removal, was found to induce PAH fragmentation and the release of reactive aromatic amines, raising additional safety considerations for both clients and practitioners [22]. These findings provide a mechanistic explanation for delayed inflammatory reactions, photosensitivity and potential long-term carcinogenic risk associated with black pigment formulations.

### 3.7. Integration with Modern Omics and Biokinetic Evidence

To avoid redundancy, the core mechanistic pathways described across studies into oxidative stress, NF-κB/MAPK-mediated inflammatory signalling, apoptotic and genotoxic responses, and lymphatic redistribution are referenced collectively hereafter unless new mechanistic insight is presented.

Future trends in molecular toxicology and omics-based risk assessment increasingly emphasise multimodal data integration, including spatial transcriptomics, single-cell sequencing, high-resolution mass spectrometry imaging and multi-omics network modelling. These approaches enable fine-scale mapping of pigment–cell interactions, pigment biotransformation, immunological microenvironments and long-term tissue remodelling. Emerging platforms also incorporate machine learning-assisted exposure modelling and probabilistic risk estimation, allowing the prediction of pigment behaviour under varying physiological conditions. Collectively, these advanced techniques promise more accurate, mechanism-driven hazard identification and more precise regulatory thresholds for pigment safety [13].

The early synchrotron and PAH-focused studies align mechanistically with recent omics-based investigations, which consistently report oxidative stress, inflammatory signalling pathway activation (NF-κB, MAPK) and wound-healing gene programme modulation following pigment exposure [8]. Together, these data support a multi-factorial toxicity model wherein particle size, chemical composition, photo-reactivity and biological persistence jointly influence pigment safety profiles.

Importantly, these mechanistic insights complement human biokinetic data, demonstrating that while soluble ink constituents may undergo cutaneous first-pass metabolism, insoluble nanoparticles and PAH-associated residues persist and redistribute via lymphatic routes [6,13]. This distinction is critical for refining risk assessment frameworks beyond simple oral exposure replacement assumptions.

**Figure 3:**
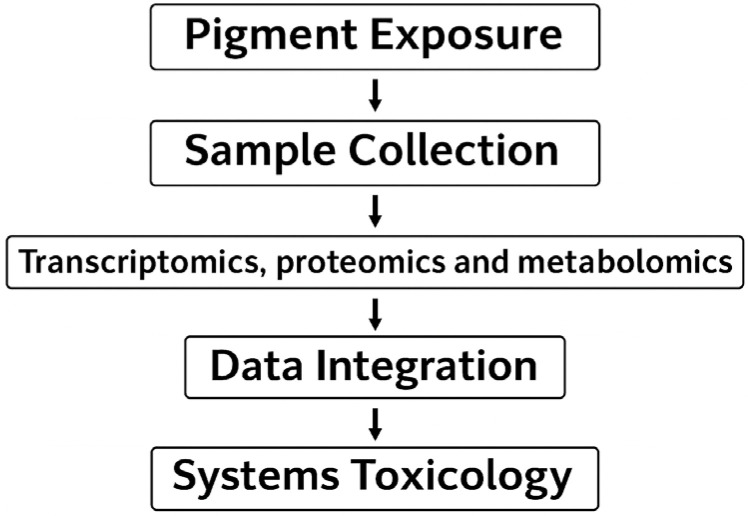
An integrated systems toxicology framework.

Figure 3 illustrates an integrated systems toxicology framework used to characterise biological responses to pigment exposure across multiple molecular layers. Following pigment exposure and structured sample collection, transcriptomic profiling captures early gene expression changes associated with stress responses, inflammation and tissue repair processes. These transcriptional signals are complemented by proteomic analyses, which identify downstream alterations in protein abundance and pathway activation; and metabolomic profiling, which reflects functional biochemical perturbations and metabolic reprogramming. Integrating these multi-omics datasets enables data-driven pathway mapping and network analysis, providing a holistic view of pigment-induced biological effects and supporting mechanistic inference within a systems toxicology context [1,2].

Recent studies published between 2024 and 2025 have further strengthened the scientific basis for evaluating pigment safety. These include reviews highlighting emerging toxicological evidence, regulatory developments and health risk surveillance relating to tattoo inks and permanent make-up. These publications contextualise the growing concerns around carcinogenic risk, pigment biotransformation and long-term adverse outcomes associated with dermal pigment exposure [31,32,33,34]. Their findings reinforce the need for a mechanistic, omics-informed risk assessment framework and support the relevance of the present systematic review in addressing modern safety challenges within pigment implantation practice.

Collectively, these findings demonstrate that pigment implantation is not a purely localised cosmetic intervention, but a biologically active process characterised by particle-size-dependent migration, chemical-specific toxicity and persistent immunological engagement. Evidence from synchrotron-based imaging, PAH toxicology, omics-enabled skin models and human biokinetic studies converges on shared mechanistic pathways, including oxidative stress, inflammatory signalling, lymphatic redistribution and the long-term cellular persistence of insoluble pigment fractions. These mechanisms provide a biologically plausible basis for the clinical reaction spectrum, immune modulation and regulatory concerns identified in this review and underscore the relevance of translating molecular-level insights into evidence-informed practitioner safeguards, pigment selection and occupational health measures within Somatology and Somatic therapy practice. The following discussion integrates these mechanistic data with regulatory frameworks and clinical considerations to outline how emerging molecular evidence can inform safer-by-design pigment use and risk-adaptive professional protocols [1,2,3,4].

## 4. Discussion

This review demonstrates that pigment implantation constitutes a biologically active and systemically relevant exposure, rather than a purely localised cosmetic procedure. Evidence spanning nanoparticle toxicology, PAH chemistry, immune modulation, biokinetics and omics-based skin models consistently indicates that pigment safety is governed by particle size, chemical composition, persistence and biological interaction, with clear implications for professional practice and occupational exposure.

Clinical hypersensitivity patterns observed in permanent make-up and cosmetic tattooing align with these mechanistic findings. The disproportionate involvement of modern red pigments and cadmium-containing yellow pigments is coherent with type IV T-cell-mediated hypersensitivity, hapten formation and granulomatous foreign body reactions surrounding persistent pigment particles. These immune archetypes plausibly explain delayed clinical presentations months to years after implantation and are supported by transcriptomic and ex vivo data demonstrating sustained inflammatory signalling and impaired resolution [8,18,19,20,21,22].

The convergence of early synchrotron-based studies, PAH phototoxicity research and recent omics-enabled skin models reinforces a multi-layered hazard framework. Insoluble nanoparticles are shown to migrate via lymphatic pathways and persist intracellularly within immune cells, while PAH-rich black pigments exhibit photochemical reactivity and genotoxic potential under UV or laser exposure. Together with human tracer studies demonstrating skin-specific first-pass metabolism of soluble components, these findings underscore the inadequacy of risk models that rely solely on oral exposure analogues [5,6,13,15,18,19,20].

### 4.1. Occupational Health and Safety Implication

**Figure 4:**
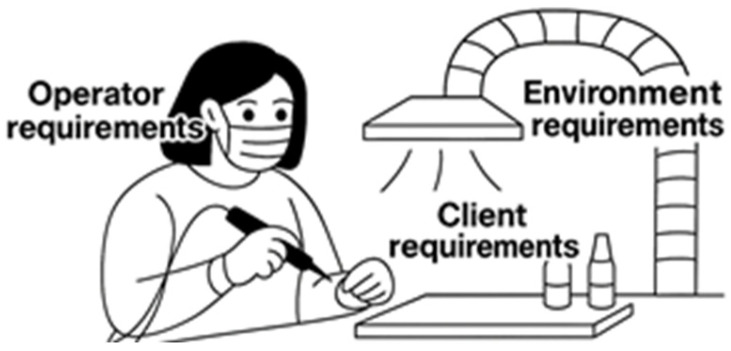
Collated occupational health and safety measures relevant to pigment implantation.

Occupational health considerations and practice-level recommendations are closely interconnected, as both arise directly from the mechanistic toxicology and exposure pathways outlined in this review. Pigment implantation procedures (Figure 4) expose both clients and practitioners to metals, nanoparticles, volatile carriers and potential microbial contaminants [7,11,12]. These exposures may occur through dermal contact, the inhalation of aerosolised particles, improper ink handling, insufficient ventilation or inadequate sterilisation practices. Recognising occupational safety as an extension of the same exposure biology that underpins client risk is therefore essential for safe and evidence-based Somatic therapy practice.

### 4.2. Practice Translation for Somatology and Somatic Therapy

**Figure 5:**
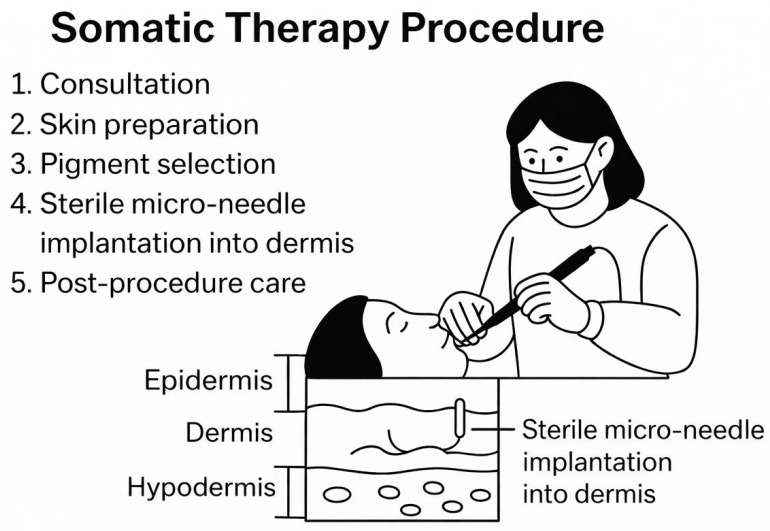
Semi-permanent make-up protocol for Somatic therapy practice.

Embedding molecular toxicology within Somatology and Somatic therapy practice enables the translation of mechanistic insights into risk-adaptive professional protocols. Figure 5 depicts the schematic that illustrates the sequential procedural steps, including consultation, skin preparation, pigment selection, sterile micro-needle implantation into the dermis and post-procedure care, together with the targeted anatomical layers of the skin. The figure highlights controlled dermal pigment deposition within an evidence-informed clinical workflow that explicitly addresses delayed reactions and lymphatic migration. Such practice translation recognises that regulatory compliance alone does not eliminate risk and that clinic-level safeguards remain essential.

### 4.3. Regulatory Context and Integration

**Figure 6:**
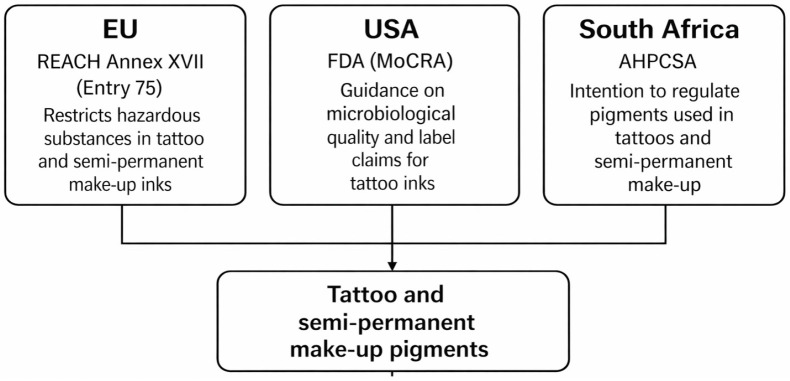
A conceptual integration of mechanistic evidence contrasts regulatory approaches across jurisdictions.

Regulatory frameworks such as REACH Annex XVII Entry 75 and the FDA’s post-MoCRA guidance represent substantial advances in pigment oversight and microbial quality control. However, enforcement data and market surveillance continue to reveal composition and labelling non-compliance, underscoring gaps between regulatory intent and product availability [9,10,11]. Figure 6 contrasts regulatory approaches across jurisdictions governing pigments used in tattooing and semi-permanent make-up, highlighting differences in chemical restriction, labelling requirements and enforcement mechanisms. The diagram illustrates how regulatory frameworks align to varying degrees with emerging toxicological and molecular evidence, including nanoparticle behaviour and chemical-specific risks. This comparison provides a context for understanding how regulatory oversight influences safer-by-design pigment selection and professional practice standards.

### 4.4. Practice-Focused Recommendation

To minimise practitioner and client risk, evidence-informed occupational health and safety (OHS) measures should be embedded into every stage of the pigment implantation procedure. These measures include the use of appropriate personal protective equipment (PPE) such as masks, gloves and protective eyewear alongside validated sterilisation and surface disinfection protocols. Local exhaust ventilation systems are essential to reduce the inhalation of aerosolised pigments and volatile solvents. In addition, environmental controls including adequate lighting, the use of single-use sterile equipment, and proper waste disposal remain critical for preventing cross-contamination and reducing cumulative occupational exposure.

Practice-focused recommendations extend these OHS principles into routine professional behaviour. Pigment selection should prioritise products with transparent ingredient disclosure and verified regulatory compliance, particularly given the increased risks associated with red azo pigments and cadmium-containing yellow formulations. Client consultations should incorporate screening for hypersensitivity, medical contraindications and inflammatory risk factors, accompanied by informed consent that explicitly addresses delayed reactions, lymphatic migration and photo-reactivity. Structured aftercare instructions and clear referral pathways further support safe outcomes.

For practitioners, ongoing training in mechanistic toxicology, ink composition and regulatory developments strengthens risk-adaptive decision-making and reinforces evidence-based clinical practice.

Taken together, the findings support several practice-relevant recommendations. Pigment selection should prioritise products with transparent compositions and documented regulatory compliance, with red azo pigments and cadmium yellow formulations treated as higher-risk in susceptible individuals [9,10,18]. Procedural safety must include validated sterilisation and device hygiene protocols aligned with microbial guidance [1,7]. Client counselling should explicitly address the possibility of delayed hypersensitivity, lymphatic migration and photo-reactivity, reinforced by structured aftercare and referral mechanisms [5,19]. Finally, robust OHS controls including PPE and adequate ventilation are essential to minimise cumulative practitioner exposure [1,27].

This review has several limitations. Substantial heterogeneity across experimental models including cell-based assays, ex vivo explants and clinical observations limited quantitative synthesis and precluded meta-analysis. Epidemiological associations between tattoo exposure and malignancy also remain inconclusive due to residual confounding and insufficient longitudinal data. Furthermore, temporal gaps between regulatory implementation and manufacturing reform may influence current market compositions and exposure profiles [7,8,9,10,14].

## 5. Conclusions

This systematic review demonstrates that pigment implantation represents a biologically active and systemically relevant exposure, rather than a purely localised cosmetic intervention. Evidence from molecular toxicology, nanoparticle research, synchrotron-based imaging, PAH chemistry, omics-enabled skin models and human biokinetic studies consistently shows that pigment safety is governed by particle size, chemical composition, photo-reactivity, persistence and immune system interaction [1,2,3,4,5]. Across experimental platforms, pigment exposure converges on shared mechanistic pathways, including oxidative stress generation, NF-κB and MAPK inflammatory signalling, apoptotic and genotoxic responses, lymphatic redistribution and long-term intracellular persistence of insoluble pigment fractions [2,3,6,7].

The integration of transcriptomic, proteomic and metabolomic data provides a system-level understanding of these processes, revealing pigment-specific inflammatory trajectories and wound-healing gene programmes that differ according to pigment colour, formulation and particulate properties [8,9,10]. Such molecular signatures strengthen causal inference and support the development of reproducible biomarkers for biocompatibility profiling and safer-by-design pigment formulation. Importantly, these mechanistic insights align with clinical observations of delayed hypersensitivity reactions, granulomatous inflammation and pigment migration, and provide a biologically plausible basis for ongoing regulatory concern and enhanced market surveillance [1,5,11].

From a translational perspective, these findings highlight the limitations of compliance-based risk management alone. Embedding mechanistic toxicology and omics-derived evidence into Somatology and Somatic therapy practice enables risk-adaptive pigment selection, improved client counselling, informed consent and procedural safeguards that explicitly address both immediate and delayed adverse outcomes [9,12]. In parallel, occupational health considerations, including aerosolised particle exposure and microbial contamination, should be interpreted as extensions of the same exposure biology and mitigated through the evidence-informed use of personal protective equipment, ventilation and aseptic technique [13,14].

In conclusion, advancing pigment safety requires the continued integration of mechanistic toxicology, a multi-omics systems approach and responsive regulatory frameworks. This convergence offers a next-generation risk assessment paradigm that enhances consumer and practitioner protection while supporting the professionalisation and recognition of Somatology and Somatic therapy within the broader health sciences [8,10,15,18]. Future research should prioritise longitudinal human studies, standardised omics endpoints and the translation of molecular markers into regulatory decision-making and clinical best practice.

## Figures and Tables

**Table 1:** Characteristics of studies included in the qualitative synthesis.

Study [Ref]	Year	Study Model	Study Design	Pigment Type	Key Findings
[1]	2021	Regulatory/review	Systematic review	Iron oxides, azo dyes	Identified safety concerns related to pigment composition and long-term exposure
[2]	2023	Human cell line	In vitro	TiO_2_ nanoparticles	Induced oxidative stress and inflammatory signalling pathways
[3]	2022	Human tissue/omics	Omics-based study	Mixed pigments	Revealed transcriptomic alterations related to immune activation and wound healing
[4]	2020	Animal model	Mechanistic in vivo	Chromium compounds	Demonstrated DNA damage and genotoxicity pathways
[5]	2021	Animal/human	Toxicokinetic	Tattoo pigments	Demonstrated pigment migration to regional lymph nodes

**Table 2:** Risk of bias assessment across included studies.

Study [Ref]	Selection Bias	Performance Bias	Detection Bias
[1]	Low	Low	Moderate
[2]	Low	Moderate	Low
[3]	Moderate	Low	Low
[4]	High	Moderate	High
[5]	Low	Low	Low

**Table 3:** Representative toxicological outcomes from included studies (descriptive summary; effect estimates as reported in source studies).

Study [Refs]	Study Model	Outcome Measure	Direction of Effect vs. Control	Effect Estimate (If Reported)	Notes
[2,4]	Human cell line (keratinocytes/fibroblasts)	Oxidative stress (ROS)	↑ ROS	+35% vs. control	Dose-dependent; CuO > ZnO (high dose)
[3,8]	Human tissue/Omics	Gene-expression profile	↑ Inflammatory markers	*p* < 0.05	Distinct programmes for black vs. red inks
[2,4]	Animal model	DNA damage index	↑ DNA damage	2.5-fold increase (95% CI 1.8–3.2)	Chromium mechanistic study

↑ refers to increase.

## Data Availability

No new data were created or analysed in this study. Data sharing is not applicable to this article.

## Supplementary Materials

The following supporting information can be downloaded at: https://www.mdpi.com/article/10.3390/ijms27125422/s1.

## Abbreviations

The following abbreviations are used in this manuscript:

AHPCSA	Allied Health Professions Council of South Africa
ALS	Amyotrophic Lateral Sclerosis
CI	Confidence Interval
CuO	Copper(II) oxide
DC	Dendritic cell
DNA	Deoxyribonucleic acid
DOI	Digital Object Identifier
ECHA	European Chemicals Agency
ESRF	European Synchrotron Radiation Facility
EU	European Union
FDA	Food and Drug Administration
Fe_2_O_3_	Iron(III) oxide
GRADE	Grading of Recommendations Assessment, Development and Evaluation
IL-8	Interleukin-8
IJMS	International Journal of Molecular Sciences
MAPK	Mitogen-Activated Protein Kinase
MoCRA	Modernization of Cosmetics Regulation Act
NF-κB	Nuclear Factor kappa-light-chain-enhancer of activated B cells
OECD	Organisation for Economic Co-operation and Development
OHS	Occupational Health and Safety
PAH	Polycyclic Aromatic Hydrocarbon
PMA	Permanent Make-Up
PPE	Personal Protective Equipment
PRISMA	Preferred Reporting Items for Systematic Reviews and Meta-Analyses
REACH	Registration, Evaluation, Authorisation and Restriction of Chemicals
RNA	Ribonucleic acid
ROS	Reactive Oxygen Species
SDG	Sustainable Development Goal
TiO_2_	Titanium dioxide
UK	United Kingdom
UV	Ultraviolet
ZnO	Zinc oxide
