# Supplementary material for: A Systematic Review on Molecular Toxicology and Omics-Based Risk Assessment of Pigments Used in Dermal Implantation Procedures: Implications for Somatology and Somatic Therapy Practice
Source: Int J Mol Sci. 2026 Jun 16;27(12):5422. doi: 10.3390/ijms27125422 (PMC13299748; doi:10.3390/ijms27125422)
Supplement: Supplementary file 1 [file ijms-27-05422-s001.zip › ijms-4121490-SI.pdf]

Table S1: Search Strategy

Database	Search Terms
PubMed, Scopus, Web of Science	(“Pigment implantation” "semi-permanent makeup" OR "micropigmentation") AND ("pigment toxicity" OR "heavy metals" OR "nanoparticles") AND ("omics technologies" OR "toxicogenomics")

Table S2: Data Extraction process

Study Design	Pigment Type	Toxicological effects	Omics Findings
In vitro/<br>*In vivo*/<br>Omics	Iron oxide, TiO_2, Azo dyes	Oxidative stress, genotoxicity, pigment migration	Transcriptomic and metabolomic changes
